# Supplementary material for: Age structure landscapes emerge from the equilibrium between aging and rejuvenation in bacterial populations
Source: Nat Commun. 2018 Sep 13;9:3722. doi: 10.1038/s41467-018-06154-9 (PMC6137065; doi:10.1038/s41467-018-06154-9)
Supplement: Supplementary file 1 — Supplementary Information [file 41467_2018_6154_MOESM1_ESM.pdf]

Supplementary Information | Age structure landscapes emerge from the equilibrium between aging and rejuvenation in bacterial populations

Audrey M. Proenca, Camilla Ulla Rang, Christen Buetz, Chao Shi, Lin Chao

SUPPLEMENTARY FIGURES

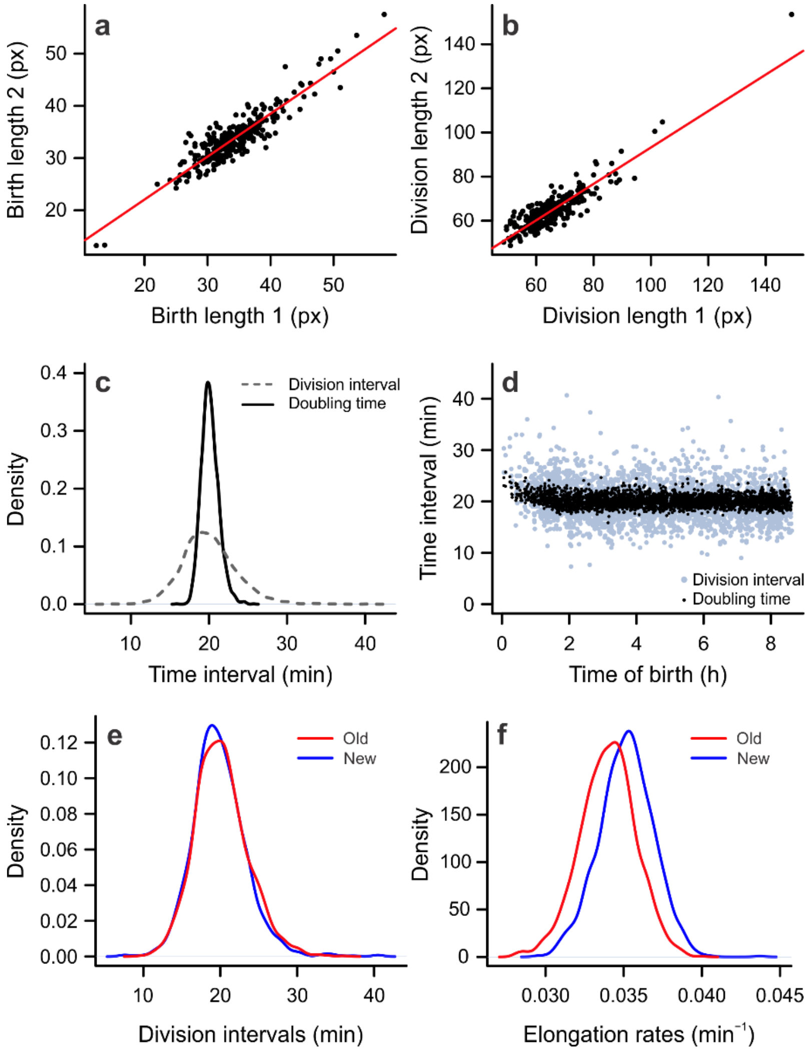

Supplementary Fig. 1. Comparison of different growth parameters and control measurements. To ensure that our measurements of bacterial length were not introducing unreasonable variability in our sample, we compared measurements acquired from mother machine cells by different individuals in separate occasions (A and B). These measurements were highly correlated for both birth lengths (A) (linear regression, $r = 0.904$, $\beta = 0.822$, $p < 0.001$) and division lengths (B) (linear regression, $r = 0.895$, $\beta = 0.825$, $p < 0.001$), thus pointing at little artificially introduced variability. Measurement errors will be part of unaccounted variability along with stochastic sources reported in this study. (C) We measured division intervals (time elapsed between birth and division) and doubling times (converted from elongation rates)

for all cells in the daughter device. Division intervals displayed much larger variance than doubling times (Bartlett test, $K^2 = 3146.9$, $p < 0.001$), despite exhibiting the same mean values (paired two-tailed t test, $t = 1.212$, $df = 2887$, $p = 0.225$). (D) Both doubling times and division intervals remained constant over time for our bacterial populations. (E) New and old daughters exhibited a significant, albeit very small, division interval difference (one-tailed t test, $t = 1.944$, $df = 2851.7$, $p = 0.026$). (F) The distinction between daughters becomes evident when comparing elongation rates, which combine the information of cell length and division intervals as a physiologically meaningful parameter. New daughters displayed faster growth (one-tailed t test, 17.842, $df = 2859.9$, $p < 0.001$), indicating that the physiological asymmetry arises due to faster elongation more than due to shorter division intervals. Thus, we performed the following analyses in this study using the more intuitive conversion of elongation rates into doubling times.

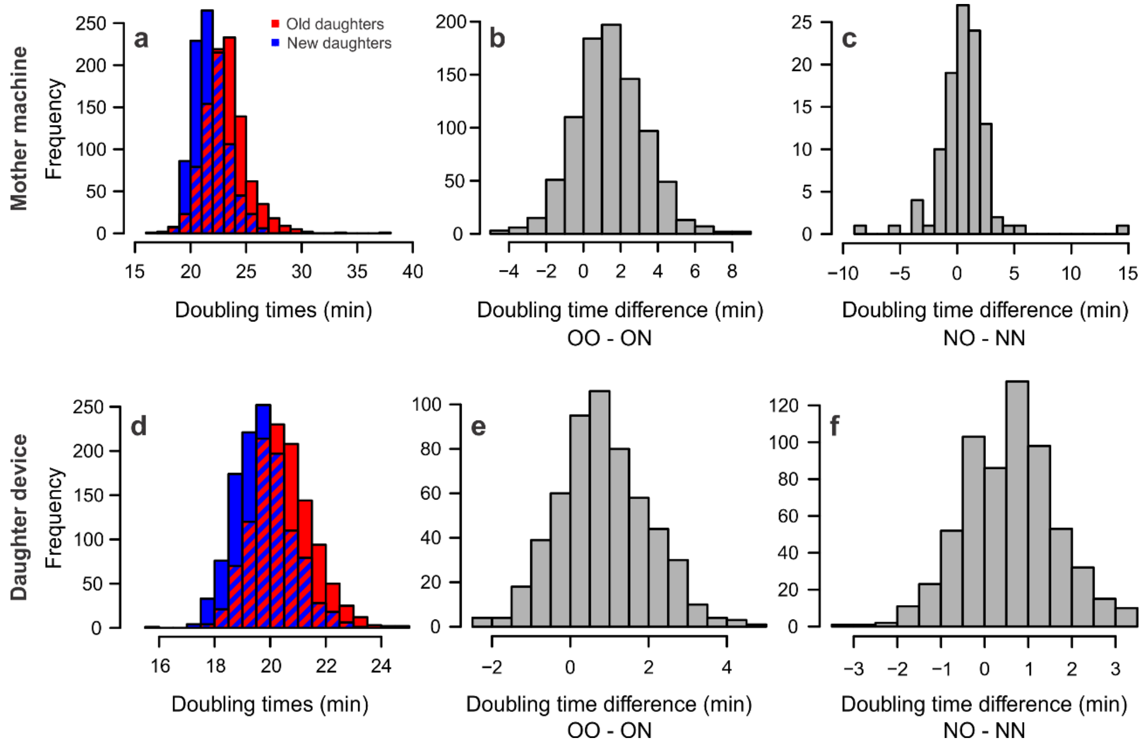

Supplementary Fig. 2. Old daughters display longer doubling times in all age categories. (A, B and C) Mother machine data corresponding to Fig. 2A. (A) Comparing all sibling pairs, old daughters displayed a significantly longer doubling time (paired one-tailed t test, $t = 21.884$, $df = 986$, $p < 0.001$). (B) Analyzing the sibling pairs at the closed edge of growth wells, we observed that the difference between OO and ON doubling times was significantly higher than zero (one-tailed t test, $t = 23.152$, $df = 881$, $p < 0.001$). (C) The same was verified for sibling pairs NO and NN, closer to the open end of the growth wells (one-tailed t test, $t = 2.308$, $df = 1.4$, $p = 0.011$). (D, E and F) Daughter device data corresponding to the same age categories. (D) Overall, old daughters displayed longer doubling times than new daughters (paired one-tailed t test, $t = 21.805$, $df = 1199$, $p < 0.001$). As in the mother machine, the difference between doubling times was significantly higher than zero for both OO-ON pairs (E) (one-tailed t test, $t = 19.219$, $df = 555$, $p < 0.001$) and NO-NN pairs (F) (one-tailed t test, $t = 13.564$, $df = 619$, $p < 0.001$).

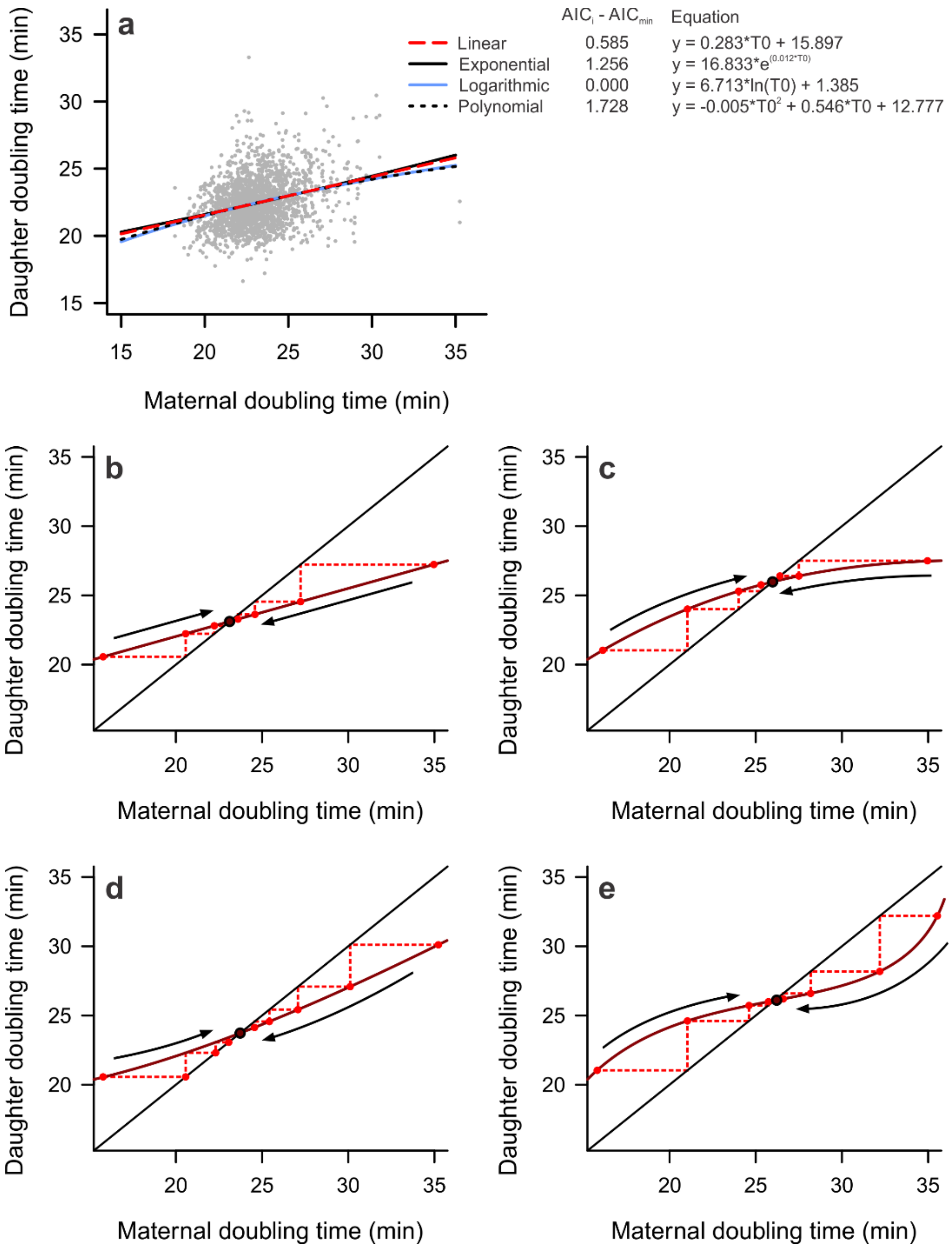

Supplementary Fig. 3. Linear and non-linear models predict stable equilibrium points. (A) Linear, exponential, logarithmic, and polynomial models showed the same positive relationship between T0 and daughter doubling times. Comparing these models through the Akaike information criterion (AIC), we observed little improvement of the fit by choosing a non-linear model. (B to E) Graphical representations of linear and non-linear models on the phase plane. The intersection between the model and identity behaves as a stable point whenever the model slope is shallower than 1 at the intersection. Thus, the existence of an equilibrium is predicted by both linear and non-linear models.

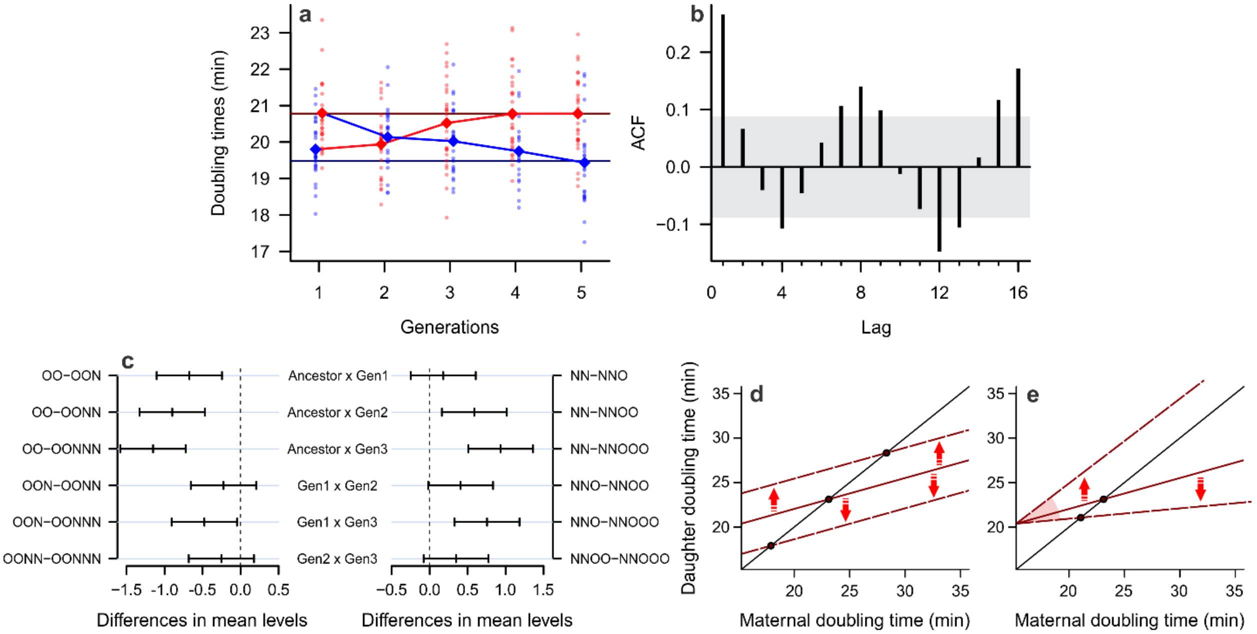

Supplementary Fig. 4. Stability of attractors for lineages away from equilibrium and in the presence of stochasticity. (A) Convergence of displaced lineages towards the correct equilibrium, expanding the visualization from Fig. 4D for one more generation. Because only complete lineages are considered, the sample size is reduced to 24 new and 30 old lineages (120 and 150 cells, respectively). (B) Autocorrelation performed for lineages from Fig. 4E cycling between equilibria through aging and rejuvenation. Lineages transition between equilibria in four generations. (C) Tukey's range test for differences of doubling time distributions for lineages transitioning from the T2 to T1 (left) or T1 to T2 (right) equilibrium (original distributions presented in Fig. 5A and 5B). (D and E) Graphical representation of the effects of stochasticity on the stability of attractors. While additive noise (represented by $b + \sigma_2$) is not disruptive (D), multiplicative noise on the slope ($a + \sigma_1$) can destabilize the attractor (E).

SUPPLEMENTARY TABLES

Supplementary Table 1. Data summary for boxplots in Fig 2A and 2B.

Device	Cell	Mean ± SD (min)	Lower whisker (min)	Lower hinge (min)	Median (min)	Upper hinge (min)	Upper whisker (min)
mother machine	OO	23.294 ± 1.783	19.104	22.084	23.173	24.230	27.404
mother machine	ON	21.849 ± 1.475	18.242	20.822	21.714	22.670	25.423
mother machine	NN	21.187 ± 1.729	18.414	20.258	20.912	21.872	24.0980
mother machine	NO	21.724 ± 2.274	18.414	20.334	21.565	22.761	25.920
daughter device	OO	20.652 ± 1.072	17.890	19.881	20.588	21.305	23.347
daughter device	ON	19.811 ± 0.988	17.671	19.157	19.772	20.427	22.316
daughter device	NN	19.604 ± 0.944	17.254	18.950	19.528	20.189	22.036
daughter device	NO	20.180 ± 0.971	17.882	19.534	20.096	20.831	22.746

Supplementary Table 2. Data summary for phase planes, Fig 3A and 4B.

Device	Daughter	Mean ± SD (min)	Equilibrium[1] (min)
mother machine	Old	23.127 ± 1.903	23.110
mother machine	New	21.778 ± 1.517	21.387
daughter device	Old	20.429 ± 1.064	20.579
daughter device	New	19.728 ± 0.986	19.525

[1]Calculated according to linear regression intersect with identity line.

Supplementary Table 3. Variance partitioning for mother machine and daughter device.

	**Mother machine**		**Daughter device**	
	**SS[1]**	**Fraction**	**SS[1]**	**Fraction**
Maternal inheritance	557.7613	0.083	406.313	0.144
Asymmetry	926.3173	0.137	295.917	0.105
Stochasticity	5253.58	0.780	2115.006	0.751
Total	6737.658		2817.236	

[1]SS = sum of squared deviations.

Supplementary Table 4. Data summary for Fig 4E.

Device	Daughter	Generation	Mean ± SD (min)
daughter device	Old	1	20.092 ± 0.051
daughter device	Old	2	20.418 ± 0.072
daughter device	Old	3	20.740 ± 0.121
daughter device	Old	4	20.779 ± 0.187
daughter device	New	1	19.946 ± 0.058
daughter device	New	2	19.831 ± 0.072
daughter device	New	3	19.574 ± 0.108
daughter device	New	4	19.435 ± 0.254

Supplementary Table 5. Data summary and statistics for distributions transitioning between equilibria. One-sample t tests verify whether the equilibrium attractors (Supplementary Table 2) represent the mean value of the doubling time distribution of each generation.

Device	Daughter	Generation	Mean ± SD	t	df	p
daughter device	OON	1	20.051 ± 0.924	4.540	61	<0.001***
daughter device	OONN	2	19.826 ± 0.871	2.783	61	0.0071**
daughter device	OONNN	3	19.573 ± 0.854	0.512	61	0.610
daughter device	NNO	1	19.985 ± 0.877	-5.845	69	<0.001***
daughter device	NNOO	2	20.393 ± 0.997	-1.717	69	0.090
daughter device	NNOOO	3	20.74 ± 1.014	1.175	69	0.244

SUPPLEMENTARY NOTES

Supplementary Note 1. Analyzing the stochasticity and stability of the doubling time equilibrium in bacterial lineages. Reproduced with permission from Dr. Massimo Vergassola, Department of Physics, University of California, San Diego, La Jolla, CA 92093

We consider the equation

$$X(n+1) = X(n)[a + \xi_1 n] + b + \xi_2(n) \qquad \text{(Supplementary Equation 1)}$$

where a and b are fixed constants and the ξ's are Gaussian independent random variables, drawn independently for each time interval, i.e. $\langle \xi_1(n)\xi_1(m)\rangle = 0$ if $n \neq m$ and $\langle \xi_1(n)\xi_1(n)\rangle = \sigma_1^2$.

We first consider the deterministic case $\sigma_1 = \sigma_2 = 0$. The iteration (1) has then the fixed point $X^* = \frac{b}{1-a}$ for $a < 1$. For $a \geq 1$, the system is unstable and X keeps growing under the iterations. For $|a| < 1$, initial deviations from the fixed point X^* relax exponentially: $\delta X(n) = \delta X(0)a^n$, where $X(n) = X^* + \delta X(n)$.

For $|a| < 1$, we next consider the additive noise case $\sigma_1 = 0$, $\sigma_2 \neq 0$. By the linearity of the iteration and the Gaussianity of ξ_2, we obtain that X will also be Gaussian. Its mean is X^* and the correlation function of its fluctuations decay exponentially with the time delay:

$$\langle \delta X(n)\delta X(m)\rangle = \frac{a^{|n-m|}}{1-a^2}\sigma_2^2 \qquad \text{(Supplementary Equation 2)}$$

In other words, the process is a discrete version of the Ornstein-Uhlenbeck process in the (time) continuum.

For $|a| < 1$, we finally consider the multiplicative noise case $\sigma_1 \neq 0$ (and for simplicity $\sigma_2 = 0$). The recurrence equation is now

$$\delta X(n+1) = \delta X(n)\big(a + \xi_1(n)\big) + \xi_1(n)X \qquad \text{(Supplementary Equation 3)}$$

The noise is now multiplying δX, which generates the non-Gaussianity of the fluctuations shown hereafter.

Multiplicative noise can lead to indefinite growth along some trajectories. Indeed, if $|a + \xi_1| > 1$ the amplitude of the fluctuations increases with the iteration, as it was shown above. The probability of $|a + \xi_1| > 1$ is unlikely if $a + \sigma_1 << 1$. However, a stretch of growing events is of course possible, with a probability that decays exponentially with the length of the stretch (this can be generally formalized using large-deviations theory). We qualitatively conclude that the far tails of the probability distribution for the fluctuations do not stabilize as they are affected by those extreme events.

More quantitatively, we can write down the equations for the moments of the fluctuations $\langle \delta X^p \rangle$ and investigate their convergence. Let us start with the variance

$$\langle \delta X(n+1)^2 \rangle = \langle \delta X(n)^2 \rangle (a^2 + \sigma_1^2) + \sigma_1^2 X \quad \text{(Supplementary Equation 4)}$$

which has the fixed point

$$\langle \delta X^2 \rangle = \frac{\sigma_1^2 X^{*2}}{1 - a^2 - \sigma_1^2} \quad \text{(Supplementary Equation 5)}$$

Note that the condition for stabilization of the variance is $a^2 + \sigma_1^2 < 1$, i.e. it is not guaranteed to hold by the condition for the stabilization of the mean $|a| < 1$.

A similar equation can be written for the third-order moment

$$\langle \delta X(n+1)^3 \rangle = \langle \delta X(n)^3 \rangle (a^3 + 3a\sigma_1^2) + 6aX^* \sigma_1^2 \langle \delta X(n)^2 \rangle \quad \text{(Supplementary Equation 6)}$$

Note that the third-order moment is generally non-vanishing, which illustrates the non-Gaussianity of the fluctuations. The condition for stabilization of the third-order moment is $a^3 + 3a\sigma_1^2 < 1$, i.e. $\langle (a + \xi_1)^3 \rangle < 1$.

The fourth-order moment obeys

$$\langle \delta X(n+1)^4 \rangle = \langle \delta X(n)^4 \rangle (a^4 + 6a^2\sigma_1^2 + 3\sigma_1^4) + 4X^* \langle \delta X(n)^3 \rangle (3a^2\sigma_1^2 + 3\sigma_1^4)$$
$$+ 6X^{*2} \langle \delta X(n)^2 \rangle (a^2\sigma_1^2 + 3\sigma_1^4) + 3X^{*4}\sigma_1^4 \qquad \text{(Supplementary Equation 7)}$$

Provided lower-order moments stabilize, the additional condition of stabilization for the fourth-order is $\langle (a + \xi_1)^4 \rangle < 1$.

The pattern for a generic order is clear: if lower-order moments stabilize, the additional condition for the stabilization of the p-th moment is $\langle (a + \xi_1)^p \rangle < 1$. These conditions are eventually going to be violated. Indeed, even for $\sigma_1 << 1$, if we consider large even orders $p = 2q$, the equality $\langle {\xi_1}^{2q} \rangle = \sigma_1^{2q}(2q-1)!!$ holds and the exponentially decaying (in q) factor σ_1^{2q} cannot compensate for the-faster-than-exponential growth of the skip factorial $(2q - 1)!!$.

In practice, for the concrete numbers $a = 0.3472$ and $\sigma_1 = 0.07$, the conditions for stabilization of the first moments $a^2 + \sigma_1^2 = 0.125$, $\langle (a + \xi_1)^3 \rangle = 0.047$, $\langle (a + \xi_1)^4 \rangle = 0.018$ are all largely satisfied. The transition to moments that do not stabilize takes place for orders in the hundreds, which are unreachable and irrelevant for any realistic measurement.
