# Supplementary material for: Age structure landscapes emerge from the equilibrium between aging and rejuvenation in bacterial populations
Source: Nat Commun. 2018 Sep 13;9:3722. doi: 10.1038/s41467-018-06154-9 (PMC6137065; doi:10.1038/s41467-018-06154-9)
Supplement: Supplementary file 3 — Description of Additional Supplementary Files [file 41467_2018_6154_MOESM3_ESM.pdf]

Description of Additional Supplementary Files

File Name: Supplementary Data 1
Description: Coordinates indicating the positioning of cells within the daughter device, and distance from the chamber meridian.

File Name: Supplementary Data 2
Description: Doubling times of mother machine lineages over generations.

File Name: Supplementary Data 3
Description: Doubling times of daughter device new and old lineages in equilibrium.

File Name: Supplementary Data 4
Description: Doubling times of daughter devices lineages transitioning between equilibria. Rows with age indicated as “old” are born from new daughters in equilibrium (Opposite1 and Opposite2) and approach the old lineage equilibrium over generations (Gen1, Gen2, Gen3).
